# Advanced Simulation Tools Applied to Materials Development and Design Predictions

**DOI:** 10.3390/ma13010147

**Published:** 2019-12-30

**Authors:** José Correia, Abílio De Jesus, Shun-Peng Zhu, Xiancheng Zhang, Dianyin Hu

**Affiliations:** 1 CONSTRUCT, Department of Civil Engineering, University of Porto, 4200-465 Porto, Portugal; 2 INEGI, Department of Mechanical Engineering, University of Porto, 4200-465 Porto, Portugal; ajesus@fe.up.pt; 3 Center for System Reliability and Safety, University of Electronic Science and Technology of China, Chengdu 611731, China; zspeng2007@uestc.edu.cn; 4 Key Laboratory of Pressure Systems and Safety, Ministry of Education, East China University of Science and Technology, Shanghai 200237, China; xczhang@ecust.edu.cn; 5 School of Energy and Power Engineering, Beihang University, Beijing 100083, China; hdy@buaa.edu.cn

**Keywords:** damage/degradation, failure mechanisms, probabilistic physics, advanced testing and statistics, materials technology, power generation systems and technologies

## Abstract

This thematic issue on advanced simulation tools applied to materials development and design predictions gathers selected extended papers related to power generation systems, presented at the XIX International Colloquium on Mechanical Fatigue of Metals (ICMFM XIX) organized at University of Porto, Portugal, in 2018. Guest editors express special thanks to all contributors for the success of this special issue—authors, reviewers, and journal staff.

## 1. Introduction

Fatigue damage represents one of the most important degradation phenomena which structural materials are subjected to in normal industrial operation, which may finally result in a sudden and unexpected failure/fracture. Since metal alloys are still the most used materials for the design of the majority of components and structures intended to carry out the highest service loads, the study of the different aspects of metals fatigue still attracts the permanent attention of scientists, engineers, and designers.

The first International Colloquium on Mechanical Fatigue of Metals (ICMFM) was organized in Brno, Czech Republic in 1968. Afterwards, regular Colloquia on Mechanical Fatigue of Metals started in 1972 also in Brno and were originally limited to participants from the countries of the former “Eastern Block”. They continued until the 12th Colloquium in 1994 at Miskolc, Hungary, every two years. After a break twelve years long, the Colloquia restarted in 2006 at Ternopil, Ukraine, followed by the ones in 2008 (Varna, Bulgaria), 2010 (Opole, Poland), 2012 (Brno, Czech Republic), 2014 (Verbania, Italy) [1], and 2016 (Gijón, Spain) [2]. The last two organizations indented to open the Colloquium to participants from all countries across Europe interested in the subject of fatigue of metallic materials [3,4]. The XIX International Colloquium on Mechanical Fatigue of Metals (ICMFM XIX) [5] was organized in 5–7 September 2018, at the Faculty of Engineering of the University of Porto, in Porto City, located at seaside in the northwest region of Portugal. This International Colloquium was intended to facilitate and encourage the exchange of knowledge and experiences among the different communities involved in both basic and applied research in the field of fatigue of metals, exploring the problem with a multiscale perspective, using both analytical and numerical approaches, without losing the perspectives of the applications [5,6,7,8].

This special issue approaches the thematic about the limits of the current generation of materials, which are continuously being reached according to the frontier of hostile environments, whether in the aerospace, nuclear, or petrochemistry industry, or in the design of gas turbines where efficiency of energy production and transformation demands increased temperatures and pressures [6]. At the same time, increasing the performance and reliability, in particular by controlling and understanding of early failures, is one key point for future materials. Moreover, increasing material lifetimes in service and the extension of recycling time are expected. Accordingly, continued improvements on “materials by design” have been possible through accurate modeling of failure mechanisms by introducing advanced theoretical and simulation approaches/tools. Based on this, researches on failure mechanisms can provide assurance for new materials at the design stage and ensure the integrity in the construction at the fabrication phase. Specifically, material failure in hostile environments occurs under multiple sources of variability, resulting from environmental load, material properties, geometry variations within tolerances, and other uncontrolled variations. Thus, advanced methods and applications for theoretical, numerical, and experimental contributions that address these issues on failure mechanism modeling and simulation of materials are desired and expected.

## 2. Scientific Topics

This issue collects selected papers from ICMFM XIX [5] related to advanced analytical and numerical simulation approaches applied to materials development and design predictions, about power generation systems. The scientific topics addressed in this issue are summarized as follows:

- Environmental assisted fatigue;
- Multi-damage/degradation;
- Multi-scale modeling and simulation;
- Micromechanics of fracture;
- Material defects evolution;
- Interactions of extreme environments;
- Microstructure-based modeling and simulation;
- Fracture in extreme environments;
- Probabilistic physics of failure modeling and simulation;
- Probabilistic optimization;
- Advanced testing and simulation;
- Life prediction and extension;
- Stochastic degradation modeling and analysis;
- Low- and high-cycle fatigue;
- Artificial intelligence methods.

## 3. Overview on the Themed Issue

This section addresses in brief the scientific papers published in this thematic issue on advanced analytical and numerical simulation approaches applied to materials development and design predictions about power generation systems.

Zhang’s group from the Harbin University of Science and Technology, Fudan University and Northwestern Polytechnical University (China) developed a fuzzy multi-extremum response surface method (FMERSM) for the comprehensive probabilistic optimization of multi-failure/multi-component structures, where the probabilistic fatigue/creep coupling optimization of turbine bladed disks was implemented—the rotor speed, temperature, and density as optimization parameters, and the creep stress, creep strain, fatigue damage, and creep damage as optimization objectives [9].

Duda, Pach, and Lesiuk [10] presented results of an experimental campaign on mechanical characterization of the AISI 304 steel with composite coatings, where the impact of the applied polyurea composite coating on selected mechanical properties, mainly, adhesion, impact resistance, static behavior, and fatigue lifetime of notched specimens was researched.

Kotch et al. [11] from the TU Dortmund University investigated the quasi-static and cyclic mechanisms to identify the possible parameters that can influence the mechanical properties of extruded chip-based profiles. In this research, the authors analyzed all specimens by X-ray computed tomography (CT) before the tests in order to be able to detect possible influences of defects like pores and delamination on the mechanical properties.

Liu et al. [12] suggested a study on a new strain prediction method by introducing intelligent algorithms—back propagation neural network (BPNN) improved by Particle Swarm Optimization (PSO). These algorithms have an important advantage in dealing with non-linear fitting and multiple input parameters. Thus, these authors have established a strain-predictive PSO-BPNN model for full-scale static experiment of a certain wind turbine blade.

Other study related to the influence of thermal–structural coupling on the blisk low-cycle fatigue life reliability analysis was introduced by Zhang et al. [13], where the generalized regression extreme neural network (GRENN) method was proposed by integrating the basic thoughts of generalized regression neural network (GRNN) and the extreme response surface method (ERSM).

Normally, fatigue crack growth relations are usually presented by using a linear-elastic stress intensity factor range, ΔK. Lesiuk [14], from the Wroclaw University of Science and Technology, has been proposed a new energy-based crack driving force for the description of the fatigue crack growth rates.

Chen et al. group from the Changsha University of Science and Technology and Guangxi University (China) presented a scientific work entitled by first-principles study on the adsorption and dissociation of Impurities on Copper Current Collector in Electrolyte for Lithium-Ion Batteries, where the stable configurations of HF, H_2_O, and PF_5_ adsorbed on Cu(111) and the geometric parameters of the admolecules were confirmed after structure optimization [15].

Luo et al. research team applied an innovative surface technology, laser shock peening (LSP), to the dual-phase TC11 titanium alloy to fabricate an amorphous and nanocrystalline surface layer at room temperature, for the ultrahigh strain-rate plastic deformation [16].

Xie et al. [17] have presented the indentation behavior, as well as the mechanical properties, of tungsten/chromium co-doped bismuth titanate ceramics sintered at different temperatures. According to this scientific work, a lower hardness and a higher fracture toughness was verified for high sintering.

Finally, a Polish–Portuguese team [18] presented an analysis of the mixed-mode (I+II, I+III) fatigue crack growth rates in bridge steel after 100-year operating time. An experimental campaign for evaluating the mixed-mode fatigue propagation behavior supported by numerical simulation was undertaken. Additionally, SEM analysis of fracture surfaces of the specimens was conducted.

## 4. Final Remarks

Guest Editors for this thematic issue are pleased with the final result of the published papers and hope that these scientific works can be useful to researchers, engineers, designers, and other colleagues involved in different thematic aspects of the advanced analytical and numerical simulation approaches applied to materials development and design predictions about power generation systems.

Additionally, the Guest Editors would like to express gratitude to all authors for their contributions and to all reviewers for their generous work that is fundamental in the dissemination of the scientific finding.

Finally, the Guest Editors would also like to express special thanks to the Editorial Board of Materials international journal for help, support, and patience and for their exceptional contributions during this time.
